# Dual-Target CAR-Ts with On- and Off-Tumour Activity May Override Immune Suppression in Solid Cancers: A Mathematical Proof of Concept

**DOI:** 10.3390/cancers13040703

**Published:** 2021-02-09

**Authors:** Odelaisy León-Triana, Antonio Pérez-Martínez, Manuel Ramírez-Orellana, Víctor M. Pérez-García

**Affiliations:** 1 Mathematical Oncology Laboratory (MOLAB), Department of Mathematics, Instituto de Matemática Aplicada a la Ciencia y la Ingeniería, Universidad de Castilla-La Mancha, Avda. Camilo José Cela, 3, 13071 Ciudad Real, Spain; odelaisy.leon@uclm.es; 2 Paediatric Haemato-Oncology Department, Hospital Universitario La Paz, 28046 Madrid, Spain; antonioperezmartinez@yahoo.es; 3 Translational Research Unit in Paediatric Haemato-Oncology, Haematopoietic Stem Cell Transplantation and Cell Therapy, Hospital Infantil Universitario Niño Jesús, 28009 Madrid, Spain; manuel.ramirez@salud.madrid.org

**Keywords:** mathematical oncology, CAR-T cells, mathematical immunology, mathematical modelling, immunotherapy of solid tumours, glioblastoma, 05.45.-a, 87.17.Aa, 87.17.Ee, 87.18.-h, 87.18.Nq, 87.19.xj, 92-10, 92C32, 92C45, 34C60

## Abstract

**Simple Summary:**

(CAR)-T cell-based therapies have achieved substantial success against different haematological malignancies. However, results for solid tumours have been limited up to now, in part due to the fact that the immunosuppressive tumour microenvironment inactivates CAR-T cell clones. In this paper we study mathematically the competition of CAR-T and tumour cells, taking into account their immunosuppressive capacity. Using computer simulations, we show that the use of large numbers of CAR-T cells targetting the solid tumour antigens could overcome the immunosuppressive potential of cancer. To achieve such high levels of CAR-T cells we propose, and study in silico, the manufacture and injection of CAR-T cells targetting two antigens: CD19 and a tumour-associated antigen. This strategy lead in our simulations to the expansion of the CAR-T cells injected and the production of a massive army of CAR-T cells targetting the solid tumour, and potentially overcoming its immune suppression capabilities. Thus, our proposed strategy could provide a way to develop successful CAR-T cell therapies against solid tumours.

**Abstract:**

Chimeric antigen receptor (CAR)-T cell-based therapies have achieved substantial success against B-cell malignancies, which has led to a growing scientific and clinical interest in extending their use to solid cancers. However, results for solid tumours have been limited up to now, in part due to the immunosuppressive tumour microenvironment, which is able to inactivate CAR-T cell clones. In this paper we put forward a mathematical model describing the competition of CAR-T and tumour cells, taking into account their immunosuppressive capacity. Using the mathematical model, we show that the use of large numbers of CAR-T cells targetting the solid tumour antigens could overcome the immunosuppressive potential of cancer. To achieve such high levels of CAR-T cells we propose, and study computationally, the manufacture and injection of CAR-T cells targetting two antigens: CD19 and a tumour-associated antigen. We study in silico the resulting dynamics of the disease after the injection of this product and find that the expansion of the CAR-T cell population in the blood and lymphopoietic organs could lead to the massive production of an army of CAR-T cells targetting the solid tumour, and potentially overcoming its immune suppression capabilities. This strategy could benefit from the combination with PD-1 inhibitors and low tumour loads. Our computational results provide theoretical support for the treatment of different types of solid tumours using T cells engineered with combination treatments of dual CARs with on- and off-tumour activity and anti-PD-1 drugs after completion of classical cytoreductive treatments.

## 1. Introduction

Chimeric antigen receptor (CAR)-T cells are modified autologous or allogeneic T cells. Their extracellular domain is engineered to recognise a tumour-associated antigen, and the intracellular domain contains a T-cell activation signal. Upon CAR engagement with the associated antigen, primary T-cell activation occurs and leads to cytokine release, cytolytic degranulation, resulting in target cell death, and T-cell proliferation [1].

CAR-T cells engineered to recognise CD19${}^{+}$ cells have been used successfully to treat B-cell malignancies. Remarkable successes have been achieved in Acute Lymphoblastic Leukaemia patients [1,2,3,4]. Furthermore, good results have also been reported for multiple myelomas [5] and diffuse large B-cell lymphomas [6], and also in refractory acute myeloid leukaemia using CD33-specific CAR-T cells [7]. These successes have motivated the study of the applicability of CAR-T cell therapies against solid tumours [8], and led to ongoing clinical trials for a variety of cancers, including glioblastomas, gastrointestinal cancers, genitourinary cancers, breast cancers, lung cancers, and others [9]. However, CAR-T cell treatments of solid tumours face significant challenges. The first is the identification of suitable tumour antigens expressed only in cancerous, rather than in healthy cells, i.e., limiting the on-target off-tumour activity of the product [10]. It is also necessary that the antigens selected for the therapy be humanised, to avoid the generation of antibodies that block the CAR-T [11]. Other major issues include T-cell persistence and expansion, T-cell trafficking into tumours, and immune resistance mechanisms that may define the ultimate fate of CAR-T cells [12].

For these reasons, it is essential to develop strategies to improve the effectiveness of therapy [13]. Combined CAR targetting has been explored as a way to improve antigen recognition and limit the possibility of tumour escape [14,15]. Several pre-clinical studies have evaluated the simultaneous targetting of two tumour-restricted antigens [16,17] and sequential treatments such as CAR-T cocktails [18]. One of the main multi-antigen-targetted CAR-T cell therapies under study here is that of dual CAR-T cells, where individual T cells are engineered to co-express two separate CARs specific to cognate antigens.

Mathematical models—whose role is to describe, quantify, and predict multifaceted behaviours—have the potential to help in finding optimal administration protocols, provide a deeper understanding of the dynamics, help in the design of clinical trials and more [19,20]. Mathematical models can disentangle complex systems, such as those arising in mutually interacting immunity, tumour growth, and immuno­therapy and have been used extensively for that purpose in the last few years (see e.g., the reviews [21,22,23,24,25,26,27]). Some recent mathematical modelling studies have been carried out to study different aspects of CAR-T cell therapies [28,29,30,31,32,33,34,35]. In this paper we study, in silico, using a mathematical model, the response of a solid tumour to a dual CAR-T product targetting both CD19 and a tumour-associated antigen. Our idea, to be explored computationally, is to use B-cells expressing the CD19 antigen to amplify the CAR-T population in a patient, which may allow for substantially higher levels of CAR-T cells to attack the tumour, thus helping to overcome the tumour’s immunosuppressive capabilities.

In this paper, we will take glioblastoma (GBM) as a specific example, but the concept explored here could be applied to different cancer types without substantial modification. Different tumour antigens that have been targetted in CAR-T clinical trials in GBM include IL13Rα2, EGFRvIII, and Her2 [36]. The main obstacles for CAR-T therapies in GBM are antigen escape due to tumour immune suppression, heterogeneous expression of identified tumour antigens, and toxicity problems [32,37].

The results of trial studies with IL13Rα2 on GBM are encouraging with regard to safety and penetration of CAR-T cells [38,39]. Persistence of CAR-T cells was observed in that study, as was the fast increase in endogenous immune cells and inflammatory cytokines after each infusion. Also, a study with CAR-T cells targetting EGFRvIII showed transient expansion of CAR-T cells, and trafficking to the brain and regions of active GBM [40].

In this paper, we will describe and study two minimal mathematical models describing the response of a solid tumour (GBM) to two different CAR-T cell-based treatment strategies. The first will describe the effect of a CAR-T targetting a tumour antigen in the presence of immune suppression. The second will describe the response to dual CAR-T cells with one of the CAR groups targetting the tumour antigens and the other targetting CD19, in order to achieve an off-tumour amplification of the product within the patient.

Our plan in this paper is as follows. First, Section 2 presents the mathematical models to be used throughout the paper and discusses how they can be parametrised. Next, Section 3 sets out the results of our computer simulations of the situation where CAR-T cells are injected bearing a single CAR group targetting an immunosuppressive tumour. Simulations of the outcome of therapy with CAR-T cells using dual CAR groups, targetting the tumour antigen and an off-tumour antigen (CD19), may be found in Section 4. Finally, Section 5 discusses the implications of the results and Section 6 summarises our conclusions. Some theoretical results on the properties on the model equations are presented in Appendix A and Appendix B.

## 2. Mathematical Models

### 2.1. Model of Solid Tumour Response to a CAR-T Cell Treatment in the Presence of Immune Suppression

The first mathematical model to be used in this paper describes the competition between a tumour population T(t) and CAR-T cells C(t), neglecting spatial aspects and other components of the immune system. In this model we assume that CAR-T cells would be amplified only at the tumour site, provided the tumour antigen is specific enough, and thus C(t) would describe the CAR-T cell population in the tumour areas. The equations of our model read

(1)dCdt=ρCCTgT+T−α1CTgC+C−1τCC,(2)dTdt=ρTT−α2CT.

The first term in Equation (1) accounts for the stimulation of CAR-T cell proliferation after encounters with tumour cells with a rate constant $\rho_{C}$ and a typical saturation population on the order of $g_{T}$ [27]. The second term describes the inactivation of CAR-T cells by tumour cells, with a maximal inactivation rate $\alpha_{1}$ per tumour cell, and a typical cellular saturation level around $g_{C}$ CAR-T cells. The last term in Equation (1) describes the natural death (or inactivation) of activated CAR-T cells. Equation (2) describes the dynamics of tumour cells, with the first term accounting for the net growth rate (with coefficient $\rho_{T}$) and the second accounting for tumour cell killing by the CAR-T cells with a rate $\alpha_{2}$. In this approach $\rho_{T}$ measures the difference between the tumour proliferation rate and any natural tumour cell death.

The parameter $\alpha_{1}$ describes the strength of CAR-T cell inactivation by the tumours. There are many mechanisms leading to T-cell dysfunction in solid tumours. Altered signalling pathways in tumour cells help produce a suppressive tumour microenvironment enriched by inhibitory cells. Metabolic constraints to cell function and survival shape tumour progression and immune cell function. In the face of persistent antigen, chronic T-cell receptor signalling drives T lymphocytes to a functionally exhausted state [41]. However, in spite of these difficulties, immune checkpoint blockade (e.g., anti-PD-1, anti-PD-L1, or anti-CTLA-4), designed to amplify endogenous anti-tumour T-cell responses, has revolutionised cancer treatment [42,43]. In 2011, ipilimumab, the first antibody blocking an immune checkpoint (CTLA-4) was authorised. This was rapidly followed by the development of monoclonal antibodies targetting PD-1 (pembrolizumab and nivolumab) and PDL1 (atezolizumab and durvalumab). The success of this approach was notable in melanoma and non-small-cell lung cancers that often contain numerous genetic mutations [44]. Today, anti-PD-1/PD-L1 antibodies are among the most widely prescribed anticancer therapies and are used as single agents or in combination with chemotherapies as first or second lines of treatment for about 50 cancer types. In line with their mechanisms of action in this paper we will assume that immune checkpoint blockade therapies will have a direct effect on $\alpha_{1}$ by reducing its value, although the exact reduction is very difficult to quantify. These drugs are not expected to have a major effect on survival as monotherapies in glioblastoma [45]; however they could have synergistic effects with the CAR-T cells as will be discussed later.

In Equation (2) we choose an exponential model to describe glioblastoma growth. This is a standard model, found to be valid for describing this type of tumour growth kinetics [46] and has the advantage of having only one adjustable parameter. In addition, it can properly describe tumour relapse from an infiltrative disseminated tumour. More complex growth models can also describe the limited experimental data available [46], and others have recently been proposed to be in better agreement with new metabolic and longitudinal growth data [47]. However, for the analysis described in this paper, we will keep the simplest form given by Equation (2).

Appendix A contains some mathematical results on the existence, uniqueness and positiveness of solutions of Equations (1) and (2) as well as on the stability of its critical points.

### 2.2. Modelling CAR-T Cells Targetting On-Tumour and Off-Tumour Antigens

A second model to be used in this paper accounts for CAR-T cells with dual CAR groups targetting two different antigens. As an example, for the case of GBM the tumour-associated antigen could be IL13Rα2, which is associated with poor prognosis and is over-expressed in >60% of those tumours, but not on normal brain tissue [48]. This antigen will be assumed to be present homogeneously in the population of tumour cells. The second antigen will be expressed by a normal tissue, whose elimination would be assumed to be compatible with life. In this paper we will think of this second antigen as being CD19, expressed by B cells. However, the same ideas should be applicable to other antigens from a normal cell population whose eradication does not compromise patient survival.

When the therapy is delivered intravenously, CAR-T cells will be initially amplified upon their encounters with CD19${}^{+}$ cells in peripheral blood and in the bone marrow, and will also trafficking to the tumour sites.

Let C(t), $\bar{C}\left(t\right)$, B(t) and T(t) be non-negative time-varying functions considering the number of CAR-T cells away from the tumour site, CAR-T cells at the tumour site, normal cells expressing the second antigen (in our case, B cells) and tumour cells, respectively. A simplified set of equations describing the dynamics of these populations is

(3)dC¯dt=ρC¯C¯BgB+B−1τCC¯−kC¯,(4)dBdt=−αBBC¯−1τBB,(5)dCdt=kC¯+ρCCTgT+T−α1CTgC+C−1τCC,(6)dTdt=ρTT−α2CT.

Equations (3) and (4) describe the off-tumour interaction between CAR-T and B cells as in previous studies [33]. The first term in Equation (3) represents B-cell induced CAR-T proliferation. The second term represents natural cell death, where $\tau_{C}$ is the activated CAR-T lifespan. Finally, the term $-k\bar{C}$ represents the trafficking of CAR-T cells to brain areas having active GBM cells, where 0<k<1 is the average fraction of CAR-T cells crossing the blood-brain barrier (BBB) and infiltrating the tumour site.

The CAR-T effect on B-cell growth is included in this model through the term $-\alpha_{B}B\bar{C}$ in Equation (4), which represents the rate of CAR-T cell induced B-cell death. The mean lifetime of B cells is described by $\tau_{B}$ in the last term of Equation (4). In the framework of our simplified approach and in line with other modelling studies we will not include a source term for newborn B-cells in the bone marrow. This is a very good approximation when dealing with the short term dynamics after the injection of the CAR-T cells, since new cell production would be orders of magnitude smaller than B-cell death. It has been hypothesised [33], that the continuous production of B-cells from CD19${}^{-}$ progenitors could lead to the maintenance of a reservoir of CAR-T cells in the bone marrow. This could have an additional positive effect in preventing relapse in B-cell malignancies, but would have no substantial effect in the context studied in this paper, since it is highly unlikely that these small populations could migrate to the brain and have any effect on relapse in malignancy.

Equations (5) and (6) are inspired in the Kuznetsov model [49] and describe the response of effector cells to the growth of tumour cells. The CAR-T cells that reach the tumour region, described by Equation (5), are stimulated by target cells T(t). The stimulation rate $\rho_{C}CT/\left(g_{T}+T\right)$ takes into account the increase in CAR-T proliferation due to encounters with tumour cells, and has a maximum value of $\rho_{C}$ as *T* gets large. CAR-T cells are killed or inactivated by tumour cells T(t) with a rate $\alpha_{1}$ and are assumed to have a finite lifespan $\tau_{C}$. Tumour cells (Equation (6)) proliferate with a rate $\rho_{T}$ and die from encounters with CAR-T cells with a rate $\alpha_{2}$.

Thus, the biological effects governing the dynamics of CAR-T cells in this mathematical model are: migration to the tumour site, stimulation by the antigens, natural cell death, and inactivation by the tumour cells. The sum of $\bar{C}\left(t\right)$ and C(t) represents the total number of CAR-T cells at time *t*.

The theoretical study of existence and uniqueness of solutions of Equations (3)–(6), together with some results on the stability of the critical points, are presented in Appendix B.

### 2.3. Parameter Estimation

The models described have several parameters to be estimated. The maximum mitotic rate $\rho_{C}$ and $\rho_{\bar{C}}$, related to the stimulation effect of the T cells by the interaction with the targets (CD19 or tumour antigen), will depend on the properties of the CAR-T product. These parameters will be taken in the range 0.2–0.9 day${}^{-1}$ according to the values reported in other models [33,50] and in agreement with the fact that stimulated CAR-T cells can undergo a few mitotic divisions per day. For current CAR-T products the mean lifetime $\tau_{C}$ of activated CAR-T cells is in the range of 1–4 weeks [51]. To estimate the tumour inactivation rate, we relied on the inhibitory role of PD-1 in immune responses [52]. A biologically broad range of values has been explored for the maximum tumour inactivation rate $\alpha_{1}$ in the range 0.01–0.99 day${}^{-1}$. This number has been estimated from tumour growth data in previous studies. For instance, the *c* parameter in Ref. [53] gives roughly $c=10^{-11}$ day${}^{-1}$ cell${}^{-1}∼\alpha_{1}/g_{C}$, which leads to a maximum value of $\alpha_{1}∼0.05$, taking, for $g_{C}$, the typical levels of T cells in blood. The value of $\alpha_{1}$ would be substantially smaller, by a factor between 10 and 10${}^{3}$, under the action of anti-PD-1 treatments [54,55]. The biochemical process of T-cell inactivation by tumour cells could be much faster. Larger values could also be possible biologically, however, we will assume that for tumours with very high immunosuppressive capabilities, PD-1 inhibitors could be used as adjuvant treatment to take $\alpha_{1}$ into the range of values studied [56].

Glioblastomas are fast-growing malignant primary brain tumours with proliferation rate $\rho_{T}$ on the order of several weeks, but have considerable variation in growth rates between individual patients [46]. Thus, we will take $\rho_{T}$ to be in the range 0.001–0.2 day${}^{-1}$.

We assume that CAR-T cells have similar killing efficiency against both the tumour ($\alpha_{2}$) and CD19${}^{+}$ cells ($\alpha_{B}$), with values around 10${}^{-11}$ day${}^{-1}$ [57]. B-cell lymphocyte lifetime $\tau_{B}$ is known to be about 5–6 weeks [58]. We will assume that in dual therapy, CAR-T cells are injected after lymphoid depletion treatment to promote expansion of CAR-T by stimulation with B cells, as usual. We set the initial number of B lymphocytes to be 2.5 × $10^{10}$ to account for the effect of this treatment [33].

Finally, the values of $g_{T}$ and $g_{B}$ indicate the inflection points from which the rate of stimulation of CAR-T cells increases, and are related to the antigen levels. These values have been estimated in previous studies by adjusting the data in experiments with mice [49]. In our case, because of the lack of experimental results on the dual CAR-Ts proposed here, they were estimated using the Equations (1) and (2) and the results obtained in Ref. [50] (stimulation rate, the maximum of transgenic copies of tisagenlecleucel, and the time of peak expansion of CAR-T cells). To do so, we neglected the immune suppression term, which is not present in leukaemias, and the parameters related to the type of cancer were chosen as in Ref. [33]. Values of $g_{T}$ and $g_{B}$ around 1–2 × $10^{10}$ cell were obtained.

A summary of the model parameters and their numerical values is given in Table 1.

## 3. Results (I): Therapy Outcomes under Immune Suppression Using CAR-T Cells with a Single CAR Group Targetting a Tumour Antigen

### 3.1. A High Level of Immune Suppression Prevents In-Patient Expansion of CAR-T Cells

Firstly, we studied the effect of the immunosuppressive strength of tumour cells as measured by $\alpha_{1}$, on the dynamics of model Equations (1) and (2). Note that in Equation (1), the term proportional to $\alpha_{1}$ represents CAR-T cell growth inhibition. When immune suppression is neglected, i.e., $\alpha_{1}=0$, an initial condition $C_{0}$ can always be found such that the treatment leads to an initial reduction of the total number of tumour cells, i.e., T(t) would initially decrease, allowing for tumour control over long times. Using Equation (2) and the condition dT/dt<0 it is easy to find that the condition for the therapy to be initially effective is $C\left(t\right)>\rho_{T}/\alpha_{2}$.

**Figure 1:**
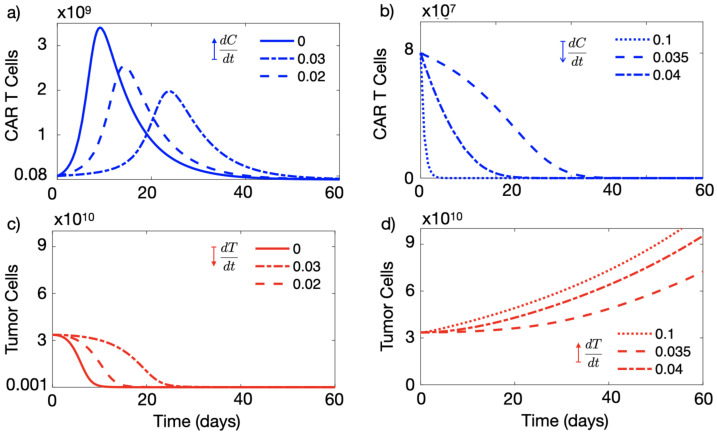
Tumour immune suppression governs the expansion of CAR-T cell sin silico. Dynamics of the number of CAR-T (blue curves) and tumour cells (red curves) governed by Equations (1) and (2) in different immune suppression scenarios. Subplots (**a**,**c**) show the results for $\alpha_{1}=0$ (solid lines), $\alpha_{1}=0.02$ day${}^{-1}$ (dashed lines) and $\alpha_{1}=0.03$ day${}^{-1}$ (dash-dotted lines) and subplots (**b**,**d**) for $\alpha_{1}=0.035$ day${}^{-1}$ (dashed lines), $\alpha_{1}=0.04$ day${}^{-1}$ (dash-dotted lines) and $\alpha_{1}=0.1$ day${}^{-1}$ (dotted lines). Initial data used in the simulations $C_{0}=8\times 10^{7}$ cells, $T_{0}=3.35\times 10^{10}$ cells and parameter values $\tau_{C}=7$ days, $\rho_{C}=0.9$ day${}^{-1}$, $\rho_{T}=1/50$ day${}^{-1}$, $\alpha_{2}=2.5\times 10^{-10}$ day${}^{-1}$ cell${}^{-1}$, $g_{T}=10^{10}$ and $g_{C}=2\times 10^{9}$.

Figure 1a,c provides an example of an effective therapy in the absence of tumour-mediated immune suppression. An initial dose of $C_{0}=8\times 10^{7}$ CAR-T cells sufficed to reduce the tumour load below observable limits in a few weeks.

Next, we studied the tumour response to CAR-T cell infusion in the presence of tumour immune suppression, i.e., for values of $\alpha_{1}>0$. Tumour control was also obtained for small $\alpha_{1}$ values (see Figure 1a,c), where the CAR-T population overcame the immune suppression and grew, promoting the death of a large number of tumour cells. The expansion of the CAR-T cell population was slower than for $\alpha_{1}=0$ and the reduction of the tumour load also occurred on longer time scales, but tumour control was also achieved in this situation, with low $\alpha_{1}$ values corresponding to tumours with low immunosuppressive capability.

We can also see in Figure 1b,d that when the value of the immune suppression parameter was increased beyond the threshold $\alpha_{1}>0.03$, the tumour and CAR-T cell dynamics changed substantially. In that situation, CAR-T cells could not expand in vivo and no longer controlled the disease, and the tumour continued growing after treatment infusion.

**Figure 2:**
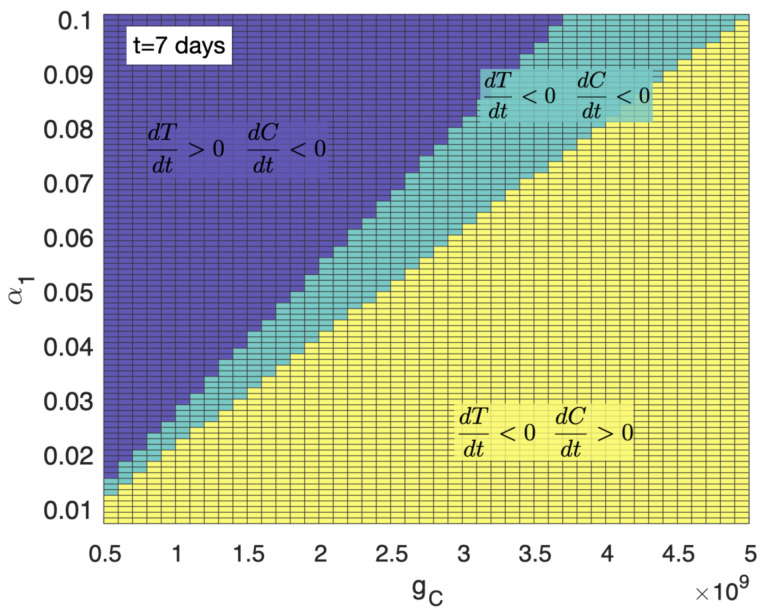
Parameter regions of control of the tumour growth dynamics seven days after infusion as a function of $\alpha_{1}$ and $g_{C}$. Yellow areas show the parameter areas in which dT/dt<0,dC/dt>0, i.e., tumour was reducing its size and CAR-T cell population increasing after seven days. Green areas are those in which dT/dt<0,dC/dt<0 after seven days, thus the tumour mass was reducing its size but the CAR-T cell was being destroyed by the cancer cells’ immune suppression corresponding to transient effect of the therapy. Purple regions are those in which dT/dt>0,dC/dt<0. Thus, the tumour was increasing its size and the CAR-T cell population decreasing after seven days, leading to therapy failure. Initial data used in the simulations were $C_{0}=8\times 10^{7}$, $T_{0}=3.35\times 10^{10}$ and parameter values $\tau_{C}=7$ days, $\rho_{C}=0.9$ day${}^{-1}$, $\rho_{T}=1/50$ day${}^{-1}$, $\alpha_{2}=2.5\times 10^{-10}$ day${}^{-1}$ cell${}^{-1}$, $g_{T}=10^{10}$.

The threshold of $\alpha_{1}$, below which the tumour dynamics were controlled by the treatment, was also found to be dependent on the value of the saturation parameter $g_{C}$, as shown in Figure 2.

Our choice of seven days to study the response was motivated by the observations of Ref. [38] where CAR-T cells were detected in the CSF after each intraventricular administration for a maximum of seven days. Similar thresholds are obtained in our analysis for values between four and seven days.

### 3.2. Initial Number of CAR-T Cells Injected Affects the Outcome of the Therapy

**Figure 3:**
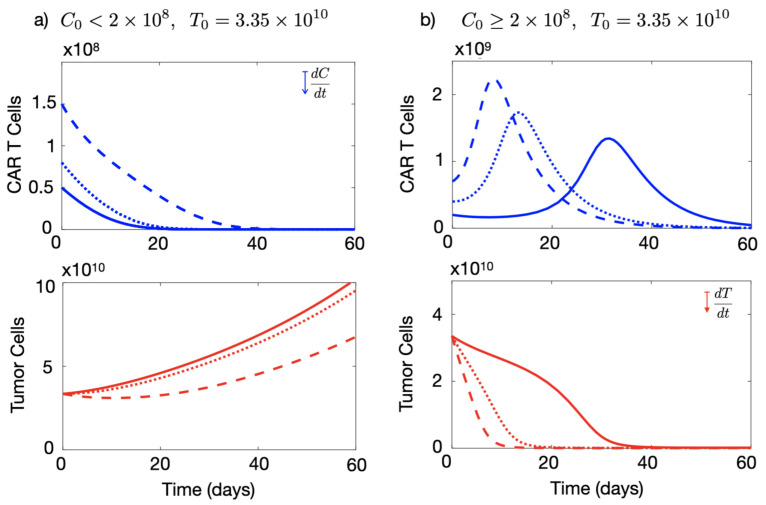
Injection of large numbers of CAR-T cells allows immune suppression effects of solid tumours to be overcome in silico. Longitudinal dynamics of the total number of CAR-T (blue) and tumour cells (red) ruled by Equations (1) and (2). The curves correspond to different values of CAR-T cells injected into a patient bearing a number of $T_{0}=3.35\times 10^{10}$ tumour cells. (**a**) CAR-T cell dynamics for $C_{0}=5\times 10^{7}$ (solid line), $C_{0}=8\times 10^{7}$ (dotted line), $C_{0}=1.5\times 10^{8}$ (dashed line). (**b**) Longitudinal dynamics of the CAR-T cells for $C_{0}=2\times 10^{8}$ (solid line), $C_{0}=4\times 10^{8}$ (dotted line) and $C_{0}=7\times 10^{8}$ (dashed line). The parameter values used in the simulations were $\tau_{C}=7$ days, $\rho_{C}=0.9$ day${}^{-1}$, $\rho_{T}=1/50$ day${}^{-1}$, $\alpha_{1}=0.04$ day${}^{-1}$ cell${}^{-1}$, $\alpha_{2}=2.5\times 10^{-10}$ day${}^{-1}$ cell${}^{-1}$, $g_{T}=10^{10}$ and $g_{C}=2\times 10^{9}$.

Next, we studied the effect of the number of CAR-T cells initially injected on the system’s dynamics for the case of CAR-T cells targetting only the on-tumour antigen. To do so, we performed an extensive number of simulations of Equations (1) and (2) over the biologically feasible range of parameters. We found a dependence of the dynamics on the number of injected CAR-T cells. Results shown in Figure 3 present some examples for numbers of cells initially injected ranging from $5\times 10^{7}$ to $7\times 10^{8}$ cells.

There were two different types of dynamics in the tumour response depending on the initial choice of $C_{0}$. For the parameters listed in Figure 3, there was a qualitative change in the dynamics around $C_{0}=2\times 10^{8}$ cells. Thus, small doses of CAR-T cells led only to a small reduction in the tumour load (Figure 3a), while for doses larger than this threshold, the therapy was able to control tumour growth in silico (see Figure 3b). The threshold was found to be related to the particular choice of parameter values and would change under different conditions.

This dynamic differs from what happens in leukaemias in which the outcome does not depend on the number of cells injected [33]. This is mainly due to the immune suppression capabilities of solid tumours included in our model equations.

### 3.3. Injection of a Large Number of CAR-T Cells Could Allow for Cure or Prolonged Tumour Control in the Presence of Immune Suppression

**Figure 4:**
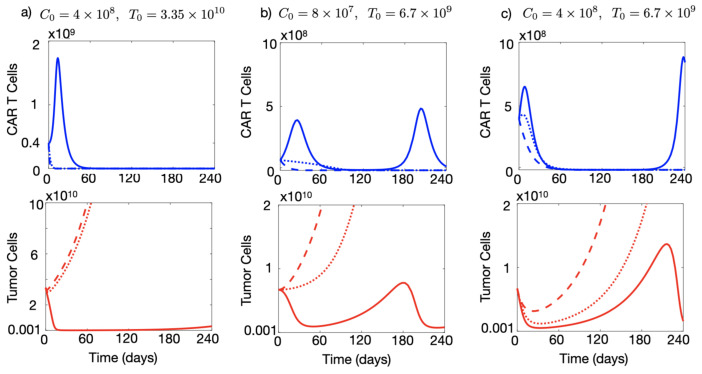
Simulated tumour and CAR-T dynamics under different initial conditions for the number of injected cells and initial tumour load. Dynamics of the number of CAR-T cells (blue curves) and tumour cells (red curves) governed by Equations (1) and (2) in three different scenarios of immune suppression: $\alpha_{1}=0.04$ day${}^{-1}$ (solid lines), $\alpha_{1}=0.07$ day${}^{-1}$ (dotted lines) and $\alpha_{1}=0.1$ day${}^{-1}$ (dashed lines). Parameter values used in the simulations $\tau_{C}=7$ days, $\rho_{C}=0.9$ day${}^{-1}$, $\rho_{T}=1/50$ day${}^{-1}$, $\alpha_{2}=2.5\times 10^{-10}$ day${}^{-1}$ cell${}^{-1}$, $g_{T}=10^{10}$ and $g_{C}=2\times 10^{9}$.

Next, we used the mathematical model as a tool to tackle the problem of tumour immune suppression against CAR-T and explored different CAR-T cell treatment strategies in silico. As a first test, we increased the dose of the CAR-T product with respect to that used in Figure 3 to $4\times 10^{8}$ cells injected. Figure 4a shows that in that situation and with a tumour immune suppression rate of $\alpha_{1}=0.04$ day${}^{-1}$ it was possible to obtain a significant reduction in the number of tumour cells, of more than four orders of magnitude, lasting for six months, which could either be compatible with cure or could provide a window of opportunity for the application of other therapies.

However, the same figure shows that higher levels of immune suppression ($\alpha_{1}=0.07$ day${}^{-1}$ and $\alpha_{1}=0.1$ day${}^{-1}$) led to the failure of the therapy and a continuous increase in the population of cancerous cells. Higher doses of CAR-T would have to be injected at these rates of immune suppression to achieve control of the disease. Figure 6c shows that for $\alpha_{1}=0.1$ day${}^{-1}$ increasing the dose above $1.5\times 10^{9}$ cells, led again to disease control.

This means that, even with immune suppression active, achieving very high levels of CAR-T cells could allow the tumour defences to be overcome, and the tumour to be defeated. However, achieving such an initial high doses is practically unfeasible. Section 4 will discuss how to achieve those large CAR-T cell doses without having to infuse them externally.

### 3.4. A High Initial Tumour Load Favours CAR-T Cell Expansion

Surgical resection is performed regularly as an up-front therapy in different cancer types. For glioblastoma, it is part of the standard treatment as it helps to rapidly reduce mass effect and neurological symptoms. The initial tumour load plays a dual role. On the one hand, a high tumour load would favour the initial expansion of the CAR-T cells, but on the other, it may enhance tumour immune suppression. Thus the question arises of what would be the optimal approach to use CAR-T cell treatments in combination with surgical resections.

To shed some light on the question, we computationally explored the idea of using CAR-T cells after performing a partial surgical resection of the tumour, a frequent situation in the context of brain tumours. In that scenario one would start treatment with a hypothetical first-line therapy with CAR-T cells, with a substantially smaller initial number of cancer cells. Assuming that the tumour load has been reduced to 20% of the initial one shown in Figure 4a, Figure 4b shows the dynamics of CAR-T and tumour cells for $T_{0}=6.7\times 10^{9}$ maintaining a low dose of CAR T, $C_{0}=8\times 10^{7}$. The decrease in the initial tumour load led to lower stimulation of the CAR-T cells and therapy failure even for tumours with low immune suppression capabilities ($\alpha_{1}=0.04$ day${}^{-1}$). However, tumour relapse could be controlled by the CAR-T cells in those tumours. Some additional tumour decrease was possible in the cases of greater immune suppression ($\alpha_{1}=0.07$ day${}^{-1}$ and $\alpha_{1}=0.1$ day${}^{-1}$) by increasing the initial CAR-T cell dose, as Figure 4c shows. In that case, transient tumour stabilisation was achieved lasting for several weeks, although the final outcome was the same as in Figure 4a.

## 4. Results (II): Therapy Outcomes under Tumour Immune Suppression Using CAR-T Cells with Dual CAR Groups with On- and Off-Tumour Activity

### 4.1. CAR-T Cells with Two Targets Provided Long-Time Tumour Control Advantages In Silico

**Figure 5:**
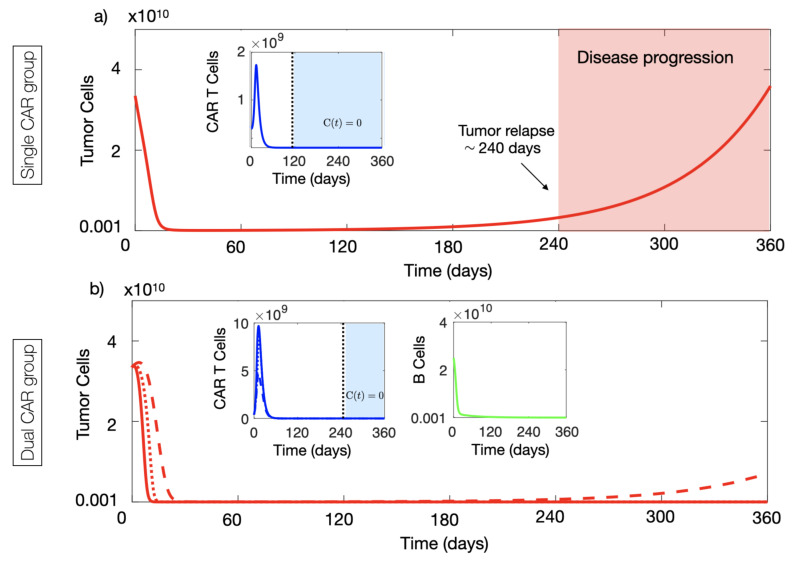
Long-term dynamics of virtual patients. (**a**) Dynamics of the number of CAR-T (blue curve) and tumour cells (red curve) governed by Equations (1) and (2). Initial conditions and tumour inactivation rate used in the simulation were $C_{0}=4\times 10^{8}$, $T_{0}=3.35\times 10^{10}$ and $\alpha_{1}=0.01$ day${}^{-1}$. (**b**) Dynamics of the number of CAR-T cells (blue curves), B cells (green curve) and tumour cells (red curve) ruled by Equations (3)–(6) in three different scenarios of immune suppression: $\alpha_{1}=0.04$ day${}^{-1}$ (solid lines), $\alpha_{1}=0.07$ day${}^{-1}$ (dotted lines) and $\alpha_{1}=0.2$ day${}^{-1}$ (dashed lines). Initial conditions used in the simulation were ${\bar{C}}_{0}=2\times 10^{8}$, $C_{0}=0$, $B_{0}=2.5\times 10^{10}$ and $T_{0}=3.35\times 10^{10}$ cells. Parameter values used in the simulations were $\tau_{C}=7$ days, $\rho_{\bar{C}}=\rho_{C}=0.9$ day${}^{-1}$, $\rho_{T}=1/50$ day${}^{-1}$, $\alpha_{2}=2.5\times 10^{-10}$ day${}^{-1}$ cell${}^{-1}$, $g_{T}=10^{10}$ cells, $g_{C}=2\times 10^{9}$ cells, $g_{B}=10^{10}$ cells, k=0.2, $\alpha_{B}=4.5\times 10^{-11}$ day${}^{-1}$ cell${}^{-1}$ and $\tau_{B}=60$ day${}^{-1}$.

We performed long-term simulations of Equations (1) and (2) with parameters as in Figure 4a and $\alpha_{1}=0.04$ day${}^{-1}$, corresponding to weak immune suppression. In this case, we observed the relapse of the disease in silico (see Figure 5a) around eight months after infusion. Tumour growth continued for several months leading to disease progression, while CAR-T cells were exhausted approximately 4 months before relapse.

However, when repeating the same simulation using model Equations (3)–(6), i.e., when using the CAR-T cells with two targets, substantially improved disease control was observed in silico. Results are summarised in Figure 5b. The interaction between the CAR-T cells in peripheral blood and the B cells stimulated the proliferation of the CAR-T cells and lead to an increased flow of these cells towards the tumour. In this case, we observe an improved expansion of the initial CAR-T cells delivered and persistence of the CAR-T product in the tumour tissue for longer times. Thus, by overcoming the tumour immunosuppressive environment, the proposed use of dual CAR-Ts could lead to improved tumour control. Figure 5b also shows the results when applying dual CAR-T therapy in more immunosuppressive tumours, where single-target therapy would have failed. Thus, the use of dual target CAR-T with on- and off-tumour activity showed substantially improved anti-tumour activity in comparison with the single-target CARs.

### 4.2. Dual CAR-T Improves the Possibility of Therapy Success

**Figure 6:**
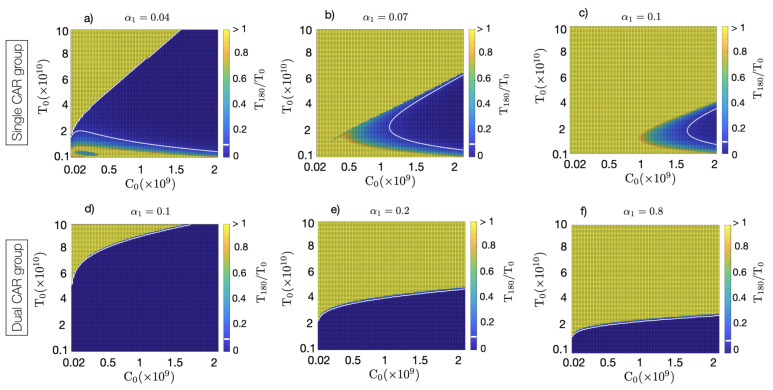
Colormap plots of the percentage of change in tumour load at six months compared to the initial load measured by the fraction $T\left(180\right)/T_{0}$, as a function of the initial number of CAR-T and tumour cells, over three immune suppression scenarios: (**a**) $\alpha_{1}=0.04$ day${}^{-1}$, (**b**) $\alpha_{1}=0.07$ day${}^{-1}$, (**c**,**d**) $\alpha_{1}=0.1$ day${}^{-1}$, (**e**) $\alpha_{1}=0.2$ day${}^{-1}$ and (**f**) $\alpha_{1}=0.8$ day${}^{-1}$. Dark blue areas, delimited by the white lines, show the initial configurations of injected CAR-T cells ($C_{0}$) and tumour loads ($T_{0}$) leading to tumour control after six months. Subplots (**a**–**c**) show the results obtained using a single CAR as governed by Equations (1) and (2). Subplots (**d**–**f**) shows results of computer simulations with the dual CARs obtained using Equations (3)–(6). Parameter values used in the simulations were $\tau_{C}=7$ days, $\rho_{\bar{C}}=\rho_{C}=0.9$ day${}^{-1}$, $\rho_{T}=1/50$ day${}^{-1}$, $\alpha_{2}=2.5\times 10^{-10}$ day${}^{-1}$ cell${}^{-1}$, $g_{T}=10^{10}$, $g_{C}=2\times 10^{9}$, $g_{B}=10^{10}$, k=0.2, $\alpha_{B}=4.5\times 10^{-11}$ day${}^{-1}$ cell${}^{-1}$ and $\tau_{B}=60$ day${}^{-1}$.

Finally, we performed a systematic study of the possibility of controlling tumour growth using single and double CAR-T therapies. Figure 6 shows the results for different values of the tumour immune suppression strength as a function of the initial number of CAR-T and tumour cells. Tumour was considered to be controlled if, after six months, the number of tumour cells was below 10% of its peak value. A threshold effect was clearly observed, with tumour control at six months being a function of $\alpha_{1},C_{0},$ and $T_{0}$. The best results were obtained for double CAR-T cell therapy, which was capable of controlling a substantially larger number of tumours according to their size and immunosuppressive capacity, with lower doses of the CAR-T product (see Figure 6d–f). For the same value of $\alpha_{1}$ (compare Figure 6c,d), dual-CAR was substantially more effective in achieving tumour control. Moreover, in situations with small initial tumour cell loads, the dual-CAR treatment was effective even for large values of the tumour immune suppression parameter, which points to a potential success of the therapy when using the treatment soon after surgery.

## 5. Discussion

After their success in treating different haematological cancers, CAR-T cell treatments have become one of the most promising novel immunotherapies in cancer. However, the transfer of their therapeutic benefits to solid tumours faces significant challenges, one of them being the immunosuppressive capabilities of the tumour microenvironment, and more specifically, of tumour cells.

In this study we constructed a mathematical model based on ordinary differential equations for the total numbers of CAR-T cells and tumour cells. This is probably the strongest assumption of our study, since tumours are complex entities having spatial structure with heterogeneous accessibility for the immune system, different types of niches and probably varying levels of immune suppression. Thus, a direct extension of this work would be to consider tumour spatial structure. The scenario of complete tumour macroscopic resection would be the one in which the mathematical model could most closely reflect the real in-patient dynamics, since spatial effects would be expected to be less relevant.

The simulations of our mathematical model suggest that the injection of a massive number of CAR-T cells could overcome the immune suppression capabilities of the tumour. The idea is simple: throw in many more T cells than the tumour can deactivate. However, this is not currently possible technically with current CAR-T products, since the number of T cells that can be obtained is orders of magnitude below the threshold for such an attack on the tumour to succeed. Moreover, only a fraction of the cells injected in the blood stream will travel to the tumour site. Although this can be partially overcome by the direct delivery of the CAR-T cells to the tumour sites these ideas have not lead to sustained therapeutical success when treating glioblastomas.

Thus, one alternative option is to generate an army of CAR-T cells within the body. For that purpose, any target allowing for the expansion of the T cells without a significant toxicity could be used. This lead us to the idea that dual-target CAR-T cells, one with on-tumour activity and other with off-tumour activity on a large population of non-essential healthy cells whose elimination does not threaten patient survival. One example of such target could be CD19 because of the large number of B-cells present in the organism, the fact that CD19 is not expressed in other tissues, and that the toxicity of current CAR-T products targetting CD19 is now well controlled.

Interestingly, our mathematical model captured the difficulties for CAR-T cell expansion when tumour immune suppression was accounted for. As in different clinical studies, the model showed that CAR-T cells targetting solid tumours have poor persistence properties, even with high doses of CAR-T. Simulations reaffirmed the relevance of the dose injected for the early outcome of the therapy. The exact threshold value that could be effective for tumour control would depend on its characteristics, and would be patient- and tumour- dependent. Immunosuppressive tumours such as glioblastoma may require higher doses of injected CAR-T cells to achieve a significant reduction in the tumour load. However, even in poorly immunosuppressive environments, the escape of the tumour due to the limiting effect of immune suppression was found to be enough to allow for relapse in the medium term.

We also explored in silico the idea of treating resected tumours with the single-CAR T cells, i.e., in scenarios of a reduction of the initial tumour load. In that case, a modest expansion of CAR-T cells was observed due to the lower levels of tumour targets. In principle, the reduction in tumour size could help in limiting the effect of tumour immune suppression. We found in silico that both processes overlapped, leading to an initial reduction in tumour size, but eventually the tumour grew back. Better results were obtained in silico for long-term tumour control when a high dose of CAR-T cells was administered to a large initial tumour. The problem is that, taking into account the reduction of cells from those injected to those travelling to the tumour region, the amount of CAR-T cells required would be too high, and thus technically unfeasible.

CAR-T with dual CAR groups targetting CD19 and the tumour antigen, would promote further stimulation of CAR-T cells through their interaction with B cells, providing a powerful source of tumour-targetting CAR-T cells. In fact, interaction with B cells is likely to occur early as they are found in blood and lymphoid organs [59]. Normal B cells would then provide a non-tumour dependent, self-renewing antigen source to support CAR-T. This double targetting, on and off the tumour, would provide a simple and pragmatic solution to improving the problem of trafficking and CAR-T cell deactivation due to immune suppression by tumour cells.

Our simple simulation model of this scenario provided substantial tumour control advantages in silico over the case of single-CAR. Substantial improvements in effectiveness were observed in cases in which CAR-T cells with a single on-tumour target had difficulty in controlling the tumour. The first situation was with highly immunosuppressive tumours, where therapy success was significantly improved by the initial boost in anti-tumour cells generated by the substantially larger initial expansion. The second situation was the one of small initial tumour loads, in which single-target CAR-T expansion would be less likely to be substantial, e.g., in cases in which an initial surgery had left only a remnant of infiltrative tumour cells. In that case, the major contribution to CAR-T cell expansion came from the CD19-bearing cells and led to the success of dual CAR therapy in silico.

That tumour control should be substantially improved using the dual CARs for low initial tumour loads is reasonable, since the CARs will be expanded off-tumour and the immune suppression capabilities of the tumour will be smaller. This means that an optimal use of this therapy would be to use it immediately after surgical resection for patients with a macroscopically complete resection. These patients have both a low initial tumour load and have at most a remnant of tumour cells that would be infiltrating the normal brain parenchyma, and so in areas where vasculature would be normal and accessible to circulating CAR-T cells.

The proposed strategy has the only limitation of the toxicity of the treatment on CD19${}^{+}$ cells. Acute toxicity is mostly related to cytokine release syndrome and neurotoxicity. These side effects of the treatment can be life-threatening in a subset of patients. However, tocilizumab and corticosteroids have been used to manage these toxicities, enabling CD19 CAR-T cells to be administered without obvious compromise in efficacy [60,61].

The PD-1/PD-L1 interaction of T cells and tumour cells leads to the inhibition of the effector function of T cells, therefore blocking this interaction has the potential to significantly enhance the anti-tumour activity of T cells and to reduce T-cell exhaustion. The combination of CAR-T with PD-1 blockade, in our case leading to reduction in the immune suppression parameter $\alpha_{1}$, is a promising strategy to enhance the effectiveness of CAR-T cells therapies [62].

Thus, our study suggests an optimal protocol for the use of these dual-target CAR-Ts with on- and off-tumour activity. Patient blood and bone marrow samples should be taken before surgery in order to start with the preparation and in-vitro expansion of the CAR-T product, and the patient should meanwhile receive surgery, and a recovery time be allowed for. Subsequently, the dual CAR-Ts should be infused, possibly in combination with anti-PD-1 treatment, and finally cytotoxic therapies (radiation therapy and chemotherapy) could be applied to kill potentially resistant cells not bearing the CAR-T tumour target. Finally, B-cell aplasia would be expected, as happens in the treatment of haematological malignancies with CD19 antigens. However, since the bone marrow is very unlikely to contain tumour cells, the sample taken initially could be used to provide an autologous bone marrow transplant after the CAR-T cells are exhausted.

## 6. Conclusions

In summary, we have constructed a mathematical model of a solid tumour response to CAR-T cells with dual targets: one of them recognising a tumour antigen and the other recognising an off-tumour antigen present in normal cells such as CD19${}^{+}$ B-cells. When only the tumour antigen was present, the therapy could overcome tumour immune suppression only when unrealistically large numbers of CAR-T cells were injected. The use of dual CARs allowed the expansion of the CAR-T population to happen even in the presence of immune suppression by tumour cells on the T-cells and allowing appropriate therapeutic levels of the T-cell population to be attained. In our simulations, this resulted in long-term tumour control, which would provide an additional tool in the fight against aggressive cancers with few therapeutic options, such as glioblastoma. We also found in silico that an optimal use of the dual-CAR-T cell therapy for glioblastoma would be to inject them immediately after extensive surgical resection and before the use of cytotoxic treatments.

In this paper we intend only to provide a theoretical proof of concept of the phenomenon. There is much work to do to explore mathematically the dynamical interplay of the different biological processes, and to find the parameter ranges best describing these phenomena. We hope that this paper will stimulate the development of experimental studies, testing the potential effectiveness of the concepts described here. If successful, CAR-T with dual targets could become a novel ingredient in combination therapies against aggressive solid tumours such as glioblastoma.

## Figures and Tables

**Table 1:** Parameter values for the Equations (1)–(6).

Parameter	Description	Value	Units	Source
	Mitotic stimulation			
$\rho_{C}$	of CAR-T cells by	0.2–0.9	day${}^{-1}$	[33,50]
	tumour cells			
	T cell concentration			Estimated
$g_{T}$	for half-maximal	10${}^{10}$	cell	from [50]
	CAR-T cell proliferation			
$\alpha_{1}$	Tumour inactivation rate	0.01–0.99	day${}^{-1}$	[53]
	CAR-T concentration			Estimated
$g_{C}$	for half-maximal	$5\times 10^{8}-5\times 10^{9}$	cell	from [53]
	tumour inactivation			
$\tau_{C}$	Activated CAR-T	7–30	day	[51]
	cell lifetime			
$\rho_{T}$	Tumour growth rate	(0.001–0.2)	day${}^{-1}$	[46]
	Killing efficiency		day${}^{-1}$	
$\alpha_{2}$	of CAR-T cells	∼ 10${}^{-11}$	× cell${}^{-1}$	[33]
	against tumour			
	Mitotic stimulation			
$\rho_{\bar{C}}$	of CAR-T cells by	(0.2–0.9)	day${}^{-1}$	[33,50]
	CD19			
$g_{B}$	B-cell concentration			Estimated
	for half-maximal	10${}^{10}$	cell	from [50]
	CAR-T cell proliferation			
$\tau_{B}$	B-lymphocyte	30–60	day	[58]
	lifetime			
	Killing efficiency		day${}^{-1}$	Estimated
$\alpha_{B}$	of CAR-T cells	∼ 10${}^{-11}$	× cell${}^{-1}$	from [57]
	against CD19${}^{+}$ cells			

**Figure A1:**
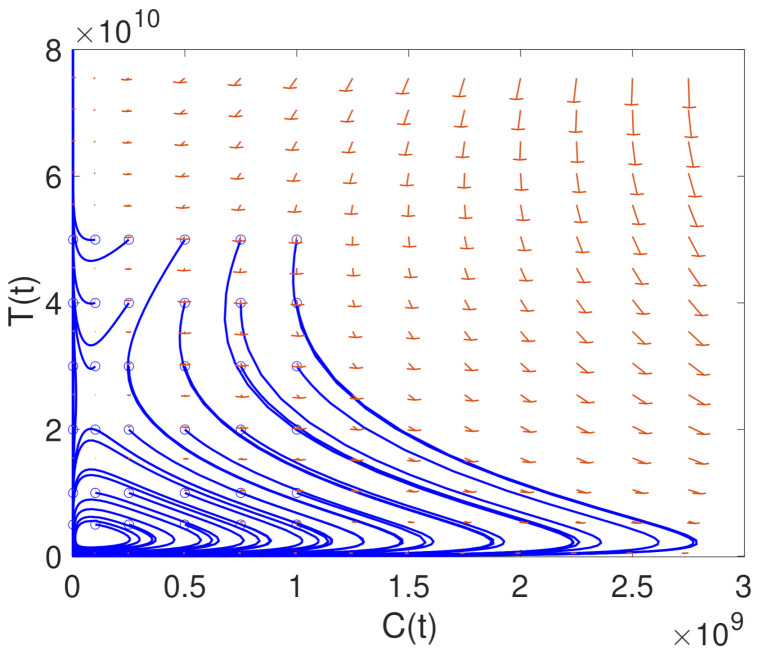
Phase space of model Equations (1) and (2). Some dynamics are represented as trajectories in the two-dimensional space (C(t),T(t)) (CAR-T cells, tumour cells) for default parameter values $\tau_{C}=7$ days, $\rho_{C}=0.9$ day${}^{-1}$, $\rho_{T}=1/50$ day${}^{-1}$, $\alpha_{1}=0.04$ day${}^{-1}$ cell${}^{-1}$, $\alpha_{2}=2.5\times 10^{-10}$ day${}^{-1}$ cell${}^{-1}$, $g_{T}=10^{10}$ and $g_{C}=2\times 10^{9}$.

## Abbreviations

CAR	Chimeric antigen receptor
GBM	Glioblastoma

## Appendix A. Basic Properties of the Mathematical Model with Single CAR Group

### Appendix A.1. Existence of Solutions

**Theorem** **A1.**
*For any non-negative initial data $\left(C_{0},T_{0}\right)$ and with all the parameters of the model being positive, the solutions to Equations (1) and (2) exist for t>0, are non-negative and unique.*

**Proof.** The ODE system (1) and (2) has bounded coefficients and the right hand side of the system is a continuous function of (C,T) in the domain $R_{+,0}^{2}$, thus the local existence of solutions follows from classical ODE theory. Since the partial derivatives of the velocity field are continuous and bounded, uniqueness follows from the Picard-Lindelof theorem. Rewrite Equations (1) and (2) as

(A1)C˙=ρCTgT+T−α1TgC+C−1/τCC,(A2)C¯=ρT−α2CT,

then we may write

(A3)C(t)=C0exp∫t0tρCT(t′)gT+T(t′)−α1T(t′)gC+C(t′)−1τCdt′,(A4)T(t)=T0exp∫t0tρT−α2C(t′)dt′,

which leads to the positivity of solutions. □

### Appendix A.2. Equilibria of the Model and Local Stability Analysis

The equilibria of Equations (1) and (2) are given by the equations

(A5)0=ρCTgT+T−α1TgC+C−1/τCC,(A6)0=ρT−α2CT,

Equation (A6) leads to either T=0 or $C=\rho_{T}/\alpha_{2}$. Using T=0 and Equation (A5) we obtain C=0. Then using $C=\rho_{T}/\alpha_{2}$ and Equation (A5) allows the quadratic expressions for T to be obtained,

(A7) $$\alpha T^{2}+T\left(\alpha g_{T}-\rho_{C}+1/\tau_{C}\right)+g_{T}/\tau_{C}=0,$$

where $\alpha =\frac{\alpha_{1}}{g_{C}+\rho_{T}/\alpha_{2}}$. The solutions of Equation (A7) are

(A8) $$T_{1,2}^{*}=\frac{-\left(\alpha g_{T}-\rho_{C}+1/\tau_{C}\right)\pm \sqrt{{\left(\alpha g_{T}-\rho_{C}+1/\tau_{C}\right)}^{2}-4\alpha g_{T}/\tau_{C}}}{2\alpha },$$

where the discriminant is non-negative and the solutions are real and positive in the case: (i) $\rho_{C}\geq {\left(\sqrt{\alpha g_{T}}+\sqrt{1/\tau_{C}}\right)}^{2}$.

The equilibrium points of the system under these conditions are

(A9) $$\begin{array}{ccc} E_{1} & = & (0,0), \end{array}$$

(A10) $$\begin{array}{ccc} E_{2,3} & = & (\frac{\rho_{T}}{\alpha_{2}},T_{1,2}^{*}). \end{array}$$

In the particular case $\rho_{C}={\left(\sqrt{\alpha g_{T}}+\sqrt{1/\tau_{C}}\right)}^{2}$, there are only two equilibria since $E_{2}=E_{3}=\left(\frac{\rho_{T}}{\alpha_{2}},\frac{1}{\sqrt{\alpha g_{T}\tau_{C}}}\right)$.

The Jacobian of the differential Equations (1) and (2) is

(A11) $$J=\left(\begin{array}{cc} \frac{\rho_{C}T}{g_{T}+T}-1/\tau_{C}-\frac{\alpha_{1}TC}{{\left(g_{C}+C\right)}^{2}} & \frac{\rho_{C}g_{T}C}{{\left(g_{T}+T\right)}^{2}}-\frac{\alpha_{1}C}{g_{C}+C}\\ -\alpha_{2}T & \rho_{T}-\alpha_{2}C \end{array}\right).$$

Let us now use Equation (A11) to study the local stability of the different equilibria given by Equations (A9) and (A10). First, for $E_{1}$ we get

(A12) $$J\left(E_{1}\right)=\left(\begin{array}{cc} -1/\tau_{C} & 0\\ 0 & \rho_{T} \end{array}\right).$$

The eigenvalues of $J\left(E_{1}\right)$ are

(A13) $$\lambda_{1}=-1/\tau_{C},\lambda_{2}=\rho_{T}.$$

thus the equilibrium point $E_{1}$ is unstable. For the other equilibrium points the Jacobian matrices are cumbersome and do not allow simple information on the stability of the equilibria to be obtained. This is why we studied the phase space of the system in the two-dimensional phase space of cancer cells and CAR-T cells. Figure A1 shows the trajectories for different initial conditions and a typical parameter choice.

In the particular case depicted in Figure A1, we computed numerically the eigenvalues of the Jacobian matrix on the three fixed points and found that $E_{1}=\left(0,0\right)$ was unstable (as expected). We obtained that $E_{2}=\left(\rho_{T}/\alpha_{2},T_{1}^{*}\right)$ had real eigenvalues, one of them positive, and thus was also an unstable point and $E_{3}=\left(\rho_{T}/\alpha_{2},T_{2}^{*}\right)$ was found to be an unstable spiral with two complex eigenvalues with a positive real part.

## Appendix B. Basic Properties of the Mathematical Model with Dual CAR Group

**Theorem** **A2.**
*For any non-negative initial data $\left({\bar{C}}_{0},B_{0},C_{0},T_{0}\right)$ and with all the parameters of the model being positive, the solutions to Equations (3)–(6) exist for t>0, are non-negative and unique.*

**Proof.** We analyse the behaviour of the vector field to prove the non-negativity of the solutions. Let $F=F\left(x\right)=\frac{dx}{dt}$ be the vector field of the system (3)–(6) with solutions $x=(\bar{C}\left(t\right),B\left(t\right),C\left(t\right),T\left(t\right))$. Starting from the positive initial condition $\left({\bar{C}}_{0},B_{0},C_{0},T_{0}\right)$, we study the direction of the vector field *F* at hyper-surfaces $\bar{C}=0$, B=0, C=0 and T=0. Let $n_{i}$ be the normal unit vector in the negative direction to plane $x_{i}=0$, for i=1,2,3,4 ( i.e., $n_{1}=\left(-1,0,0,0\right)$, $n_{2}=\left(0,-1,0,0\right)$, ..., $n_{4}=\left(0,0,0,-1\right)$) and consider the scalar products $F\cdot n_{i}$. Then, $F\cdot n_{1}=0$, $F\cdot n_{2}=0$ and $F\cdot n_{4}=0$ at hyper-surfaces $\bar{C}=0$, B=0 and T=0, respectively. Then, the hyper-surfaces $\bar{C}=0$, B=0 and T=0 are invariant. In the case of hyper-surface C=0 it is found that $F\cdot n_{3}=-k\bar{C}<0$. Hence, $R_{+,0}^{4}$ is a positive invariant domain for Equations (3)–(6). Therefore, we have proved the non-negativity of solutions $\left(\bar{C}\left(t\right),B\left(t\right),C\left(t\right),T\left(t\right)\right)$. Since the ODE system (3)–(6) has non-negative solutions and the right-hand side of the system is a continuous function of $\left(\bar{C},B,C,T\right)$ in the domain $R_{+,0}^{4}$, the existence of solutions follows form the Cauchy-Peano theorem. Moreover, the partial derivatives of the velocity field are also continuous and bounded in $R_{+,0}^{4}$. Then, using the Picard-Lindelof theorem we have proved the uniqueness of solutions. □

### Equilibria of the Model and Local Stability Analysis

We begin by calculating the fixed points and determining their stability. These are the points $E_{1}=\left(0,0,0,0\right)$ and $E_{2}=\left(0,0,\frac{\rho_{T}}{\alpha_{2}},T_{1,2}^{*}\right)$, where $T_{1,2}^{*}$ corresponds to the point previously calculated in Equation (A8). To analyse the stability of these points, we calculate the Jacobian matrix of Equations (3)–(6)

$$J=\left(\begin{array}{cccc} \frac{\rho_{\bar{C}}B}{g_{B}+B}-1/\tau_{C}-k & \frac{\rho_{\bar{C}}\bar{C}g_{B}}{{\left(g_{B}+B\right)}^{2}} & 0 & 0\\ -\alpha_{B}B & -\alpha_{B}\bar{C}-1/\tau_{B} & 0 & 0\\ k & 0 & \frac{\rho_{C}T}{g_{T}+T}-1/\tau_{C}-\frac{\alpha_{1}TC}{{\left(g_{C}+C\right)}^{2}} & \frac{\rho_{C}g_{T}C}{{\left(g_{T}+T\right)}^{2}}-\frac{\alpha_{1}C}{g_{C}+C}\\ 0 & 0 & -\alpha_{2}T & \rho_{T}-\alpha_{2}C \end{array}\right).$$

For the equilibrium point $E_{1}=\left(0,0,0,0\right)$, the Jacobian matrix is

$$J=\left(\begin{array}{cccc} -1/\tau_{C}-k & 0 & 0 & 0\\ 0 & -1/\tau_{B} & 0 & 0\\ k & 0 & -1/\tau_{C} & 0\\ 0 & 0 & 0 & \rho_{T} \end{array}\right).$$

and the eigenvalues are $\lambda_{1}=-\left(k+1/\tau_{C}\right)$, $\lambda_{2}=-1/\tau_{B}$, $\lambda_{3}=-1/\tau_{C}$ and $\lambda_{4}=\rho_{T}$. Thus, $E_{1}$ is an unstable equilibrium point. The study of the stability of $E_{2}$ is similar to its analogue in the case of Equations (1) and (2), where a simple analytical expression for the eigenvalues cannot be obtained.
